# The Development of Extra-Articular Manifestations in Children With Enthesitis-Related Arthritis: Natural Course or Different Disease Entity?

**DOI:** 10.3389/fmed.2021.667305

**Published:** 2021-05-13

**Authors:** Ilaria Pagnini, Mariangela Scavone, Ilaria Maccora, Maria Vincenza Mastrolia, Edoardo Marrani, Federico Bertini, Lovro Lamot, Gabriele Simonini

**Affiliations:** ^1^Rheumatology Unit, Meyer Children Hospital of Florence, University of Florence, Florence, Italy; ^2^Radiology Unit, Meyer Children Hospital of Florence, Florence, Italy; ^3^Department of Pediatrics, School of Medicine, University of Zagreb, University Hospital Centre Zagreb, Zagreb, Croatia

**Keywords:** enthesitis-related arthritis, extra-articular, midfoot, biologic, sacroiliitis, children, JIA

## Abstract

**Introduction:** Enthesitis-related Arthritis (ERA) is a specific category of juvenile idiopathic arthritis (JIA) characterized by axial and/or peripheral arthritis, and enthesitis, although other different extra-articular manifestations may encompass its clinical spectrum.

**Materials and Methods:** In order to examine if ERA-JIA with extra-articular involvement may represent a different entity from ERA without extra-articular involvement, we performed a retrospective, observational, monocentric study, in a cohort of ERA patients followed between 2001 and September 2020 at the Pediatric Rheumatology Unit of Meyer Children Hospital of Florence. We analyzed the demographic, clinical, laboratory and imaging data at the disease onset, as well as after 3, 6, and 12 months follow up.

**Results:** We have enrolled 53 patients, 33 males. At the time of diagnosis, average age was 10.9 years, 53 patients had active arthritis and 25 active enthesitis. The middle foot involvement was present in 20 patients. Twenty-five children achieved clinical remission on medication. Extra-articular manifestations were observed in 14 patients, of whom 3 had inflammatory bowel disease, 5 uveitis, one uveitis associated with Crohn disease, 4 SAPHO syndrome, one celiac disease. The cohort was stratified according to the presence/absence of extra-articular manifestations. It was observed that middle foot involvement was more frequent in patients with no extra-articular manifestations (18/39 vs. 2/14; χ^2^ = 4.45, *p* = 0.05). Additionally, patients presenting extra-articular manifestation needed more frequently (12/14 vs. 21/39, χ^2^= 4.45, *p* = 0.05), and preciously (months: 3.7 ± 5.4 vs. 16.7 ± 26.5, *p* = 0.02), treatment with biologic agents. Finally, these patients achieved belatedly (months: 31.6 ± 32.3 vs. 22.9 ± 18.3, *p* = 0.01) and less frequently (3/14 vs. 22/39; χ^2^= 5.50, *p* = 0.03) the clinical remission on medication. Eventually, extra-articular involvement inversely correlated with the middle-foot arthritis (ρ_s_ −0.29, *p* = 0.03), the chance to achieve remission on medication (ρ_s_ −0.31 e *p* = 0.02), as well as the chance to keep overall remission, with and without medication (ρ_s_ −0.28, *p* = 0.04).

**Conclusion:** In our cohort, children diagnosed with ERA-JIA at the onset of disease and then developed extra-articular manifestations show the absence of middle foot involvement and worse prognosis with an early need for the use of biologic agents, and overall low chance to achieve remission.

## Introduction

Enthesitis-related Arthritis (ERA) is one of the more common types of Juvenile Idiopathic Arthritis (JIA) (1), frequently affecting males after 6-year-old. In Europe and North America, ERA comprises almost 10% of JIA cases (2, 3) as opposite to certain countries in Asia where it represents the most common JIA subtype (35–40% of JIA patients) (4–7). The main findings of this particular JIA subtype are axial and/or peripheral arthritis, inflammatory back pain, enthesitis and a specific association with the HLA-B27 typing.

While the criteria of International League of Associations for Rheumatology (ILAR) are most commonly used by pediatric rheumatologists to classify this entity in children, ERA patients are often regarded as having an undifferentiated form of juvenile spondyloarthritis (jSpA) (1, 8). However, as opposite to ERA, jSpA encompasses differentiated forms such as juvenile ankylosing spondylitis (jAS), psoriatic arthritis (PsA), reactive arthritis (ReA) and arthritis associated with inflammatory bowel disease (IBD), as well (8). Moreover, different extra-articular manifestations, such as uveitis inflammatory bowel disease, celiac disease, Synovitis Acne Pustulosis Hyperostosis Osteitis (SAPHO) syndrome or less common cardiac and/or pulmonary involvement may encompass jSPA spectrum. Conversely to other JIA subtypes, uveitis in ERA patients is typically characterized by an acute, symptomatic onset with red, painful, and photophobic eye, usually unilateral and frequently recurrent (9, 10). Inflammatory bowel disease, including Crohn disease, ulcerative colitis and undifferentiated colitis, occurs in 5–10% of patients affected by ERA, more frequent in males, at the onset or during disease course (11). In addition, celiac disease occurs in 1–8% of patients with ERA (12), while SAPHO syndrome is an even less frequent complication characterized by axial involvement along with enthesitis and peripheral arthritis and typical cutaneous findings (13, 14).

Although uncommon, aortic insufficiency as well as myocarditis, endocarditis and pericarditis, often with spontaneous resolution, can occasionally occur in ERA patients (15–17). Pulmonary involvement, characterized by diminished chest expansion with decreased vital capacity, are very rare (18, 19), whilst central nervous system (CNS) diseases are seldom reported in ERA (19).

In order to define the clinical phenotype of ERA-JIA with extra-articular features, we enrolled patients affected by ERA and stratified them by the presence of extra-articular manifestations. Moreover, to address if ERA-JIA with extra-articular involvement may represent a different entity from ERA without extra-articular involvement, we compared the clinical features, laboratory data, treatment modalities, disease activity and outcome of a pediatric cohort of ERA-JIA patients.

## Materials and Methods

We performed a retrospective, observational, monocentric study, in a cohort of patients affected by ERA and followed between 2001 and September 2020 at the Pediatric Rheumatology Unit of Meyer Children's University Hospital in Florence.

All patients fulfilled the International League of Associations for Rheumatology (ILAR) criteria (1, 20) for the diagnosis of ERA-JIA. We analyzed the demographic, clinical, laboratory and imaging data at the disease onset, and thereafter at 3, 6, and 12 months follow up. All data were enrolled in a customized database, including:

- **Demographic variables:** (1) gender; (2) age at the onset of clinical manifestation; (3) age at the diagnosis, entered as the age the child met the (ILAR) criteria for ERA; (4) history of HLA-B27-related disease in first-degree relatives, including ankylosing spondylitis, ERA, inflammatory bowel disease, reactive arthritis (Reiter's syndrome), and acute anterior uveitis. Psoriasis was excluded since is a mandatory exclusion criterion for ERA diagnosis if present in patient and/or in a first-degree relatives.
- **Clinical variables:** (1) disease duration; (2) interval between disease onset and diagnosis; (3) assessment of the number and type of affected joints. Active arthritis was defined as a joint with swelling not caused by bone enlargement, or limitation of motion in combination with pain or tenderness; (4) assessment of the number and type of affected enthesis. Enthesitis was defined as discretely localized tenderness at the point of insertion of ligaments, tendon, joint capsules, or fascia to bone (21), and assessed according to the Maastricht Ankylosing Spondylitis Enthesitis Score (MASES), but including in additional plantar fascia and calcaneus enthesis (22); (5) middle foot involvement, mono or bilateral; (6) inflammatory back pain, defined according to the Assessment of Spondylarthritis International Society (ASAS), expert criteria (23), as lumbosacral spinal pain persisting at least 3 months in patients with: age <40 years, insidious onset, improvement with exercise, not improved with rest, pain at night (at least 4 of 5 requirements need to be present) (21, 23); (7) tenderness of sacroiliac (SI) joints, compression of pelvis, distraction of the SI joints by Patrick's test (21, 23); (8) limited anterior spinal flexion, assessed by the modified Schoeber test (23); (9) limited lateral spinal flexion, according to the ASAS expert criteria (23); (10) extra-articular features, at the onset or during disease course (range was referred in months): uveitis, diagnosed by slit lamp examination; inflammatory bowel disease diagnosed by endoscopy and; celiac disease, diagnosed by laboratory test (antigliadin antibody, ant-transglutaminasis antibody detected by Enzyme-linked immunosorbent assay [ELISA] and anti-endomision antibody detected by Immunofluorescence [IFI]) and by biopsy of duodenum tract; SAPHO syndrome was diagnosed by the presence of the key clinical features; (11) Disease activity measures, according to the American College of Rheumatology pediatric criteria (24); (12) Disease remission indices, according to the preliminary criteria for clinical remission in JIA (25), including no active entheses.
- **Laboratory variables:** Hemoglobin value (Hb), erythrocyte sedimentation rate (ESR), C-reactive protein (CRP), presence of the HLA-B27 allele, and anti-nuclear antibody (ANA) positivity.
- **Imaging SI assessment:** Magnetic resonance imaging (MRI) study of the SI joints: dynamic contrast-enhancement MRI before and after administration of contrast medium was performed as described (26, 27). In all patients MRI images were obtained with a 1.5 Tesla unit (Philips Intera: Philips, Eindhovenn, The Netherlands) using a pelvic array body coil with reg following sequences: semicoronal Short tau inversion recovery (STIR) sequences, semiaxial Turbo spin echo (TSE) T1-weighted sequences, semicoronal Spectral Presaturation with Inversion Recovery (SPIR) T1-weighted sequences, semiaxial TSE T2-weighted sequences, semicoronal dynamicc T1fat-saturated (FS), and semiaxial T1 SPIR after administration of intravenous gadolinium (0.1 mmol/kg). Assessment of the MRI examinations included a grading of 0–3 (0 normal, 1 minimal, 2 moderate, 3 severe) of the following findings: erosion, sclerosis (low signal intensity on T1and/or T1 FS), bone marrow edema (high signal intensity in STIR), contrast enhancement in the bone and in the joint space, and joint space narrowing and/or Widening. All assessment and grading were performed at four anatomical sites for each SI joint: the sacral and iliac sites of the cartilaginous and ligamentous portions go the joint. In addition, gadolinium contrast enhancement was performed and acute/active sacroiliitis on MRI was defined if bone marrow edema on STIR or bone marrow osteitis on T1 post-gadolinium was detected and located in subchondral or periarticular bone marrow (28). Moreover, monolateral and bilateral sacroiliitis was graded 0–4 corresponding to the New York criteria, according to Aarhus criteria accepted by Outcome Measures in Rheumatoid Arthritis Clinical Trials (OMERACT) Rheumatoid Arthritis Magnetic Resonance Imaging Scoring System (RAMRIS) (29);
- **Therapeutic variables**, as therapy administered at onset and throughout the disease course in terms of Disease-modifying anti-rheumatic drugs (DMARDs) and/or biologic treatment.

### Statistical Analysis

All results were expressed as mean and standard deviation (SD), or median and range. Mann-Whitney *U* test, Kruskal-Wallis test, Wilcoxon signed-rank test for paired samples, chi-square test (**χ**^**2**^) and Fisher exact test, when appropriate, were used to compare data. Pearson and Spearman correlation tests were used to determinate correlation coefficients for different variables (sex, age at diagnosis, number of active joints at diagnosis, number of active enthesis at diagnosis, middle foot involvement, inflammation of SI joints, increased ESR, increased CRP, ANA positivity, HLA-B27 positivity, DMARDs treatment and timing of therapy, biologic treatment and timing of therapy, remission time). Multiple stepwise regression was performed to determine variables that could correlate independently with the development of extra-articular involvement and a confirmed diagnosis of ERA at last available follow-up. The predictors used in the final model were those showing a significant correlation in the univariate analysis. Non-parametric tests were used, where necessary, due to the small size of our groups and to the skewness of data. A p level <0.05 was considered statistically significant. All analyses were performed on SPSS for MAC, version 26.0 (SPSS Inc., Chicago, IL, USA).

## Results

Fifty-three children fulfilled the criteria for ERA at the time of diagnosis and were then enrolled into the study: 33 males and 20 females with a median age at diagnosis of 10.9 years (range 3–16 years). Except for eight patients, all children were Caucasian. This represents 10% of the total cohort of patients with JIA followed in our center in the same period.

As regards family history, the presence of an autoimmune disease (Ankylosing Spondylitis, Rheumatoid Arthritis, Hashimoto thyroiditis, type 1 Diabetes Mellitus, celiac disease, vitiligo and inflammatory bowel disease) was described in 23 subjects (43.4%) of whom five reported multiple autoimmune disease.

At diagnosis, active arthritis was observed in all 53 children, whilst enthesitis, SI involvement and the middle foot in 25 (47.2%), 23 (43.3%), and 20 (37.7%) patients, respectively. Table 1 details the clinical features, laboratory parameters and therapeutic approaches in our cohort of ERA-JIA patients.

**Table 1:** Clinical features, laboratory parameters and therapeutic approaches of our cohort of diagnosed as ERA-JIA at the onset of disease.

**Clinical features**	**N of pts**	**Non-extra-articular involvement**	**Extra-articular involvement**
Arthritis at diagnosis	53	39	14
- Symmetrical involvement	18	12	6
- Asimmetrical involvement	35	27	8
- Oligoarticular	33	22	11
- Polyarticular	20	17	3
Enthesitis at diagnosis	24	20	4
- Monolateral involvement	13	12	1
- Bilateral involvement	11	8	3
**Number of enthesitis at diagnosis**			
- One	13	12	1
- Two	8	6	2
- Three	2	1	1
- Four	1	1	-
SI involvement	23	16	7
- Monolateral	18	12	6
- Bilateral	5	4	1
Middle foot involvement	20	18	2
- Monolateral	20	18	2
- Bilateral	7	7	-
**Laboratory values**			
Increased ESR	27	19	8
[mean value ± SD (mm/h)]	37.1 ± 29.6	36.1 ± 30.9	40.1 ± 26.2
Increased CRP	27	20	7
[mean value ± SD (mg/dl)]	2.26 ± 2.9	2.17 ± 3.1	2.51 ± 2.9
Anemia	12	9	3
[Hb mean value ± SD (g/dl)]	10.0 ± 1.4	9.9 ± 1.3	10.3 ± 1.2
ANA positivity	16	11	5
HLA B27 positivity	21	18	3
**Therapeutic approach**			
DMARDs	48	37	11
- Methotrexate	28	37	10
- Sulfasalazine	20	19	1
Biologic agents	33	21	12
- Adalimumab	23	14	9
- Etanercept	8	5	3
- Golimumab	1	1	-
- Abatacept	1	1	-

*SI, sacroiliac; ESR, erythrocyte sedimentation rate; CRP, C reactive protein; Hb, hemoglobin; ANA, antinuclear antibodies; DMARDs, disease modifying anti-rheumatic drugs*.

At last available follow-up (median time from disease onset 42 months, range: 4–193), 25 patients (47.2%) reached clinical remission on medication after a median time of 14 months (range 6–62). Clinical remission on medication lasted for a median time of 67 months (range 9–142). Fifteen patients (28.3%) reached clinical remission without medication for a median time of 16 months (range 6–35).

Over the disease course, 14 patients (26.4%) developed extra-articular manifestations, which were not present at diagnosis but complained during the clinical course. In particular three patients had inflammatory bowel disease, one child had acute anterior uveitis associated to IBD, five patients had uveitis, four patients had SAPHO syndrome, and one patient had celiac disease. The mean time between ERA-JIA diagnosis and the extra-articular manifestation onset was 18.8 months (range 9–60). In three subjects, extra-articular symptoms (uveitis) were concomitant at the time of ERA onset and diagnosis. Thus, once the patients developed a confirmed diagnosis of IBD, documented by typical histopathology then that affected patients have been classified as IBD-associated arthritis. The same for children at disease onset classified as ERA and later on as SAPHO syndrome due to the development of additional clinical findings. Therefore, eight (15%) children did not fulfill anymore the ERA diagnosis.

Persistent arthritis was the indication for the biologic treatment, along with the development of uveitis, IBD and SAPHO syndrome in the cohort who exhibited over the disease course extra-articular involvement. Among these 14 children, two children, one with coeliac disease and one with uveitis, did not receive biologic treatment.

Moreover, of the 23 patients affected by sacroiliitis in ERA, 13 patients were in treatment with DMARDs (12 with sulphasalazine and 1 with Methotrexate), while 10 patients were in treatment with anti-TNF alpha inhibitors. Of these 10 patients, 7 developed extra-articular manifestation. In particular: two patients developed acute anterior uveitis, one patient developed gastrointestinal involvement, one patient developed celiac disease and three patients developed SAPHO syndrome. Eventually, among 23 children with sacroiliitis, 4 out of 23 (17%) did not fulfill anymore the ERA diagnosis.

**Figure 1:**
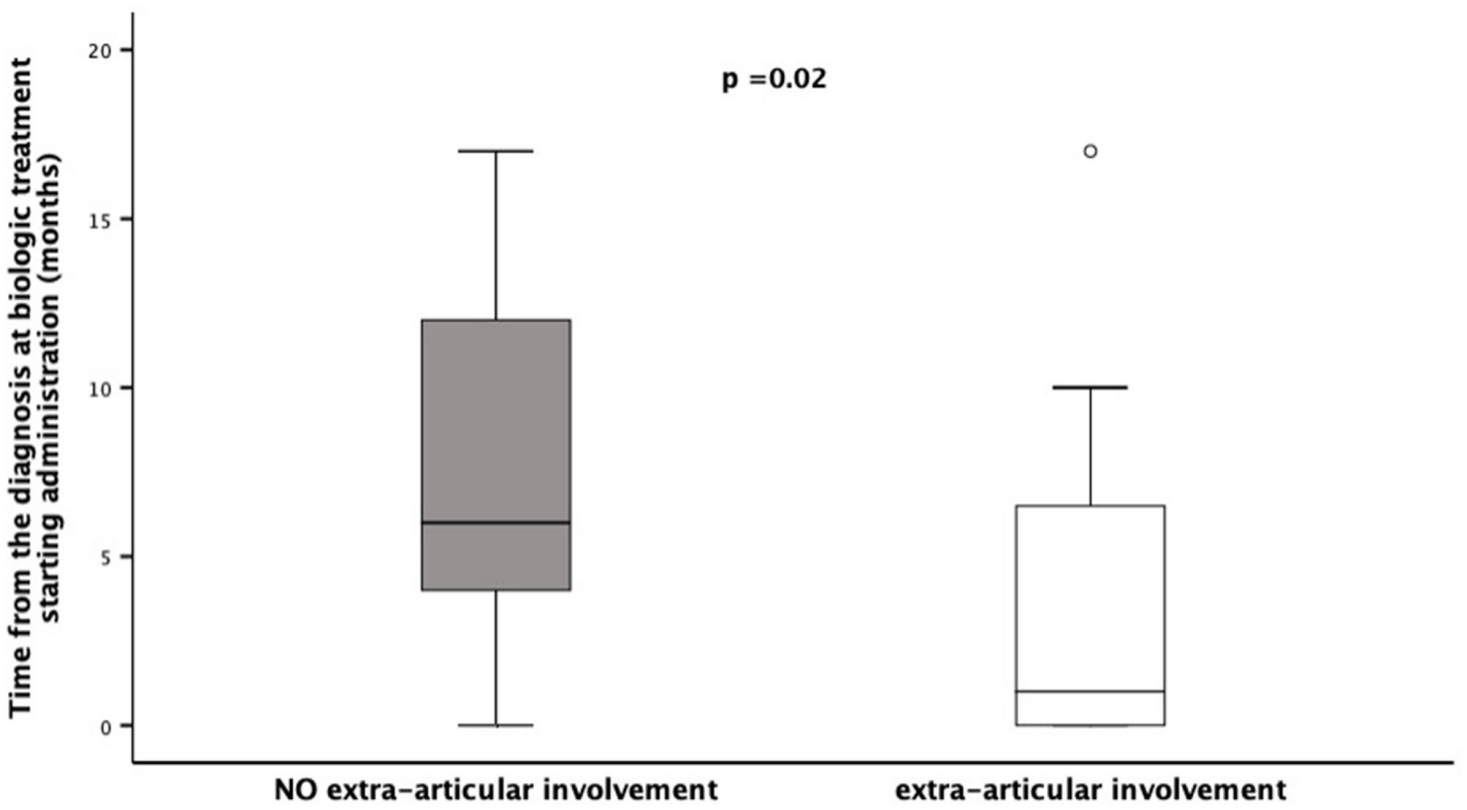
Time of Biologic Treatment starting in a cohort of 53 children diagnosed as ERA-JIA at the onset of disease. Figure shows the time from the diagnosis at biologic treatment starting administration (months) in 12 children with extra-articular involvement, diagnosed ERA-JIA at the onset (white box) and in 21 ERA-JIA children without extra-articular manifestations (gray box). The central line represents the distribution median, boxes span 25th to 75th percentiles, and error bars extend from 10th to 90th percentiles. Dots are values higher than the 90th percentile. (*p* = 0.002). ERA-JIA, enthesitis-related arthritis juvenile idiopathic arthritis.

**Figure 2:**
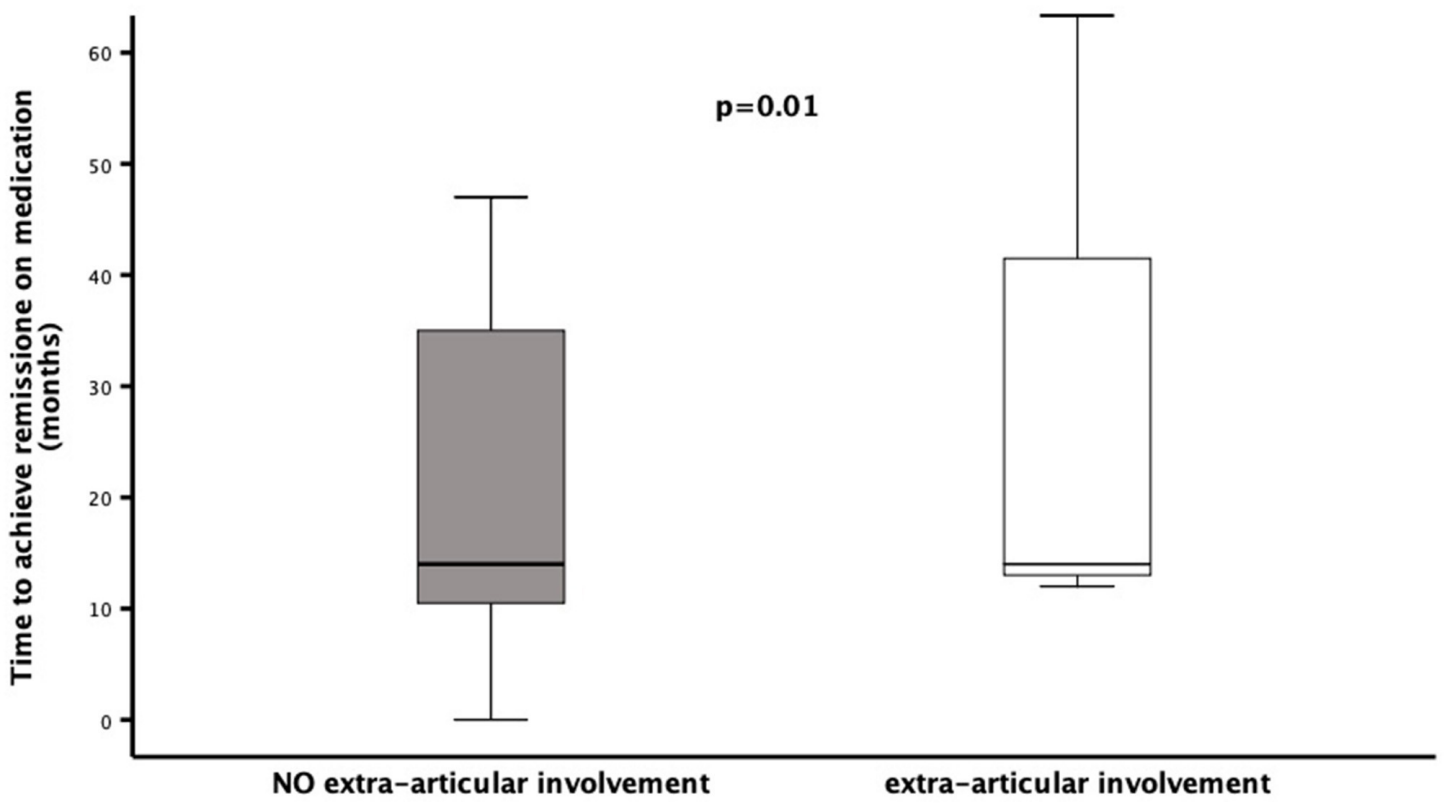
Time to reach clinical remission on medication in a cohort of 53 children diagnosed as ERA-JIA at the onset of disease. Figure shows the time to reach clinical remission on medication (months) in 3 ERA-JIA children with extra-articular involvement (white box) and in 22 ERA-JIA children without extra-articular manifestations (gray box). The central line represents the distribution median, boxes span 25th to 75th percentiles, and error bars extend from 10th to 90th percentiles. Dots are values higher than the 90th percentile. (*p* = 0.002). ERA-JIA, enthesitis-related arthritis juvenile idiopathic arthritis.

Patients' stratification according to the presence/absence of extra-articular manifestations revealed that the middle foot involvement was more frequent in patients without extra-articular manifestations (18/39 vs. 2/14; χ^2^= 4.45, *p* = 0.05). Additionally, patients presenting extra-articular manifestations needed more frequently (12/14 vs. 21/39, χ^2^= 4.45, *p* = 0.05), and preciously (months: 3.7 ± 5.4 vs. 16.7 ± 26.5, *p* = 0.02), treatment with biologic agents (Figure 1). Moreover, this group of patients achieved less frequently (3/14 vs. 22/39; χ^2^= 5.50, *p* = 0.03) and belatedly (months: 31.6 ± 32.3 vs. 22.9 ± 18.3, *p* = 0.01) the clinical remission on medication (Figure 2). Overall, considering all the children on clinical remission at last available follow-up, including patients receiving and stopped treatment, this group of patients had less chance to maintain remission (4/14 vs. 26/39: χ^2^= 6.08, *p* = 0.01) (Table 2).

**Table 2:** Comparison statistics stratifying into extra-articular- vs. non-extra-articular group.

	**Non-extra-articular involvement**	**Extra-articular involvement**	**χ^2^*P*-values**
Middle foot involvement (*n*/*N*, %)	18/39 (46%)	2/14 (14%)	4.45, 0.05
Number of children receiving biologic treatment (*n*/*N*, %)	21/39 (54%)	12/14 (85%)	4.45, 0.05
Number of children on clinical remission on medication (*n*/*N*, %)	22/39 (56%)	3/14 (22%)	5.50, 0.03
Number of children on clinical remission at last available follow-up (*n*/*N*, %)	26/39 (67%)	4/14 (29%)	6.08, 0.01

Eventually, extra-articular involvement inversely correlated with the middle-foot arthritis (ρ_s_ −0.29, *p* = 0.03), the achievement of remission on medication (ρ_s_ −0.31 e *p* = 0.02), as well the chance to keep remission, with and without medication (ρ_s_ −0.28, *p* = 0.04)

No further correlations considering the number of active joints, the number of enthesitis, the presence of SI, the elevation of inflammatory markers, ANA and HLA-B27 positivity, type and duration of treatment with DMARDs were detected. In multiple regression analysis, where the development of extra-articular involvement at last available follow-up was set as dependent variable, the middle foot involvement and the clinical remission on medication remained as negative predictors of extra-articular involvement (multiple R = 0.41, multiple adjusted R2 = 0.14, F: 5.19, *p* < 0.009).

Considering that eight children, four with IBDs and four with SAPHO syndrome, did not fulfill anymore the criteria for ERA-JIA over the disease course according ILAR classification, we additionally performed a sub-analysis limited to the 45 ERA-JIA children who fulfilled the diagnosis of ERA according ILAR criteria even over the diseases course.

The same statistical significance findings have been detected regarding the middle foot involvement: it was more frequent in patients without extra-articular manifestations (18/39 vs. 0/6; χ^2^ = 4.61, *p* = 0.03), and extra-articular involvement in ERA-JIA inversely correlated with the middle-foot arthritis (ρ_s_ −0.32, *p* = 0.03). Conversely, the others statistical significance results have not been kept.

## Discussion

The central pathogenic event in various forms of arthritis in adults and children alike is certainly chronic inflammation of the synovial tissue and joint destruction (30, 31). In the specific types of the arthritis, such as spondyloarthritis in adults and ERA subtype of JIA in children, other structures, such as enthesis and/or axial joints, can be affected by inflammation in similar fashion (11, 32). Finally, the inflammatory process can spread beyond the musculoskeletal structures throughout the body causing the various extra-articular manifestations (33). However, for reasons not entirely clear, this scenario occurs in less than quarter of patients with SpA (34), while, to the best of our knowledge, our study was the first to report the incidence of several extra-articular manifestations in a single cohort of patients with ERA-JIA.

The results of our study showed that 26% of the enrolled subjects had and/or developed over the disease course extra-articular manifestations: three IBD, five uveitis, one uveitis associated with Crohn disease, four SAPHO syndrome and one celiac disease.

Therefore, eight children, representing the 15% of this monocentric cohort, and fulfilling the criteria for ERA at the onset of disease, developed additional clinical findings and were then reclassified accordingly: IBD-associated chronic arthritis, not included in ILAR criteria, and SAPHO syndrome.

In our monocentric cohort, it appears that the current ILAR criteria failed to properly classify the 15% of patients. The complexity of childhood rheumatic diseases makes them difficult to classify in coherent set criteria, as patients may present simultaneously a various range of manifestations shared by different disorders or develop over time additional findings. Mostly in childhood, the phenotype of each patient may evolve over time and extend beyond defined schemes, creating overlapping entities challenging to systemize while relying on the present knowledge (35). Although the ILAR criteria for ERA-JIA may fit jSpA peculiarities better than other classifications utilized mainly for adults, in these set of criteria, jSpA is covered by different subtypes of JIA, namely psoriatic arthritis, ERA and undifferentiated arthritis (1). However, certain phenotypes are excluded (e.g., reactive arthritis, arthritis associated with IBD) and the axial involvement is not specifically recognize, since the ERA subtype does not discriminate between axial and peripheral disease. Indeed, how to categorize JSpA still remains a debated and unsolved issue (8, 36, 37).

Any attempt to classify SAPHO has been equally difficult, considering that the articular sings may be the first presentation of the latter cutaneous findings. While several authors consider this disorder as a group of autoinflammatory bone disorders others highlighted the strong link with SpA, especially in later life stage (38).

Since some pathognomonic manifestation often occur late in disease course, the best chance to properly identify a disease may be a close clinical monitoring over time and reassessing the diagnosis when new sings/symptoms appear.

Interestingly, there were several important differences among patients with and without extra-articular manifestations in our cohort. Stratifying our cohort according to the presence/absence of extra-articular manifestations, we identified that the midfoot involvement in our cohort of ERA-JIA patients seems to correlate with no extra-articular manifestations, which was not observed in previous studies.

However, in accordance with recent literature, the midfoot involvement seems to be one of the key characteristics of ERA-JIA and may significantly affect the ERA prognosis. Phatak et al. (39), in an elegant prospective study, reported that the midfoot disease produced important functional limitation. During the course of the last years, numerous studies addressed the midfoot involvement as characteristic feature of ERA-JIA (40–42). The inflammation of midfoot in spondyloarthritis can engage pathological processes such as tarsal swelling, synovial inflammation, bone overgrowth, enchondral ossification, enthesophytosis, bone bridging, and finally ankylosis of the tarsal bones, leading to the distinctive form of the severe involvement of the feet, named ankylosing tarsitis (43). Interestingly, the similar changes are noticed in ankylosing spondylitis (AS), which is considered the differentiated form of spondyloarthritis, as opposite to the undifferentiated forms such as ERA-JIA. Therefore, the increased frequency of midfoot involvement in ERA-JIA patients, that do not develop extra-articular manifestations, could indicate the distinctive type of inflammation which spreads mainly throughout the musculoskeletal structures, avoiding the other body systems.

When our analysis was limited to the 45 children who still meet the ILARA criteria over the disease course, only the middle foot involvement remained statistically significant related to the ERA-JIA group with no extra-articular involvement. In our opinion, this result may strengths the hypothesis that middle foot involvement strictly belongs to the ERA phenotype, since other clinical data, even those included into the ILAR criteria were not able enough to clearly differentiate a group of ERA children, who at the onset met the ILAR criteria, and were then diagnosed with another different disease.

Another important finding in our cohort was that ERA-JIA children that later developed extra-articular manifestations needed more frequently and preciously treatment with biologic agents. Moreover, these patients achieved belatedly and less frequently the clinical remission, on medication as well as without medication. Therefore, development of a different disease, such as IBD, as well as a systemic syndrome such as SAPHO, clearly modifies the prognosis and outcome in these children initially diagnosed as ERA-JIA according to ILAR criteria. Clinical experience from adult patients with AS reports a common association with extra-articular manifestations, although often subclinical (44). The presence of these comorbid conditions negatively affects the patients' quality of life and overall outcome (45). Emerging knowledge suggests that extra-articular manifestations in AS may be the expression of a unique inflammatory process involving the whole body (46). Therefore, the adoption of a therapeutical strategy that takes into account patients' symptoms in their entirety without focusing on a single area should be considered. In this perspective, an early recourse to a TNF-inhibitor (adalimumab and infliximab to be preferred above etanercept) for the management of AS with extra-articular manifestations and comorbid conditions may represent the most appropriate therapeutic approach (45–47).

According to several studies, ERA is associated with worse function, poorer quality of life, and increased pain intensity (48–50). In particular, HLA-B27 positivity, tarsitis, hip arthritis within the first 6 months, and older age of disease onset are associated with worse function, quality of life and pain (51–53). However, some of these studies reported some unexpected potential associations. Specifically, it has been reported that normal ESR correlates with a lower likelihood of attaining inactive disease (54). Interestingly, the authors suggested that TNF-alpha inhibitors may be more effective in patients whose active disease is accompanied by robust active systemic inflammation mirrored by raised inflammatory markers. Conversely, disease manifestations, such as joint pain and enthesalgia, that may not be strongly associated with systemic inflammation, tend to respond less well to TNF-alpha inhibitors. In this complicated scenario, non-randomized studies showed that adding methotrexate to TNF-alpha inhibitors seems to be more effective than TNF-alpha inhibitors alone, which could be explained by different mechanisms of these two DMARDs (55, 56). Nevertheless, whatever the reason, these findings are in favor of the concept of the widespread disease in some patients with ERA-JIA.

We fully acknowledge the limitation of our results imposed by the small sample size which limits the strength of the conclusions. However, the present cohort represents the 10% of all JIA children followed at our unit over the course of 20 years, which is the percentage reported in other cohorts as well (2, 3).

Additionally, another advocated shortcoming might be that enthesitis was not confirmed by imaging studies, thus biasing the ERA-JIA diagnosis at the onset of the disease. However, none of the enrolled patients has been classified at diagnosis as ERA-JIA only by the clinical presence of enthesis, without a concomitant peripheral arthritis. Currently, according ILAR criteria, confirmation of Enthesitis by imaging is not a required criterion.

In conclusion, based on clinical observations from our cohort of patients, we hypothesize that there could be two distinctive disease phenotypes in JIA children with ERA, depending on the presence or absence of the extra-articular manifestations. Specifically, midfoot involvement was associated with ERA-JIA diagnosis and the absence of extra-articular manifestations. Conversely, development of extra-articular manifestations over the time increases the chance of a different disease, such as Crohn disease and SAPHO syndrome, thus associated with worse prognosis, an early need for the use of biologic agents, longer time to achieve remission with and/or without medication. According to this mono-centric cohort, middle foot involvement seems to be a specific feature of JIA-ERA, since children with tarsitis, classified as ERA at the onset, still fulfilled the same criteria over the course of the disease.

While these findings might already have an important implication in the diagnostic and therapeutic approach to JIA patients with ERA, results from a prospective study involving larger multicenter cohort are mandatory for their confirmation.

## Data Availability Statement

The raw data supporting the conclusions of this article will be made available by the authors, without undue reservation.

## Ethics Statement

Ethical review and approval was not required for the study on human participants in accordance with the local legislation and institutional requirements. Written informed consent from the participants' legal guardian/next of kin was not required to participate in this study in accordance with the national legislation and the institutional requirements.

## Author Contributions

IP designed the study and drafted the preliminary paper. MS and EM collected the patients' data. IM, MM, and LL participated in drafting the paper. FB helped in imaging assessment. GS performed statistical analysis, design the study, and finalized the paper. All authors contributed in writing the papers and approved the final draft of it.

## Conflict of Interest

The authors declare that the research was conducted in the absence of any commercial or financial relationships that could be construed as a potential conflict of interest.
